# Current Status of Intestinal Stem Cell Research: Mechanisms, Intestinal Diseases, and Organoid Development

**DOI:** 10.3390/cells15151396

**Published:** 2026-08-01

**Authors:** Yanqiu Li, Rui Lai, Yue He, Kexin Cheng, Li Cheng, Jie Yi, Ying Li, Siyuan Zhou

**Affiliations:** Acupuncture and Tuina School, Chengdu University of Traditional Chinese Medicine, Chengdu 611137, China; liyanq064@stu.cdutcm.edu.cn (Y.L.);

**Keywords:** intestinal stem cell, research development, regulatory mechanisms, colorectal cancer, inflammatory bowel disease, intestinal organoid

## Abstract

**Highlights:**

**What are the main findings?**

- Intestinal stem cell (ISC) research has laid the solid groundwork for deciphering intestinal homeostasis and disease pathology.
- Intestinal organoid technology has established a robust platform for ISC research.

**What are the implications of the main findings?**

- The proliferation and differentiation of ISCs are orchestrated by diverse microenvironmental signals, and this intricate regulatory network holds promise for elucidating disease mechanisms and advancing therapeutic strategies.
- Integrating intestinal organoids with other emerging technologies can further deepen our understanding of ISC biology, offering a novel avenue for investigating pathological mechanisms and potential therapeutic targets.

**Abstract:**

Intestinal stem cells (ISCs) are essential for maintaining intestinal homeostasis and repairing injury, and have garnered extensive attention in recent years. ISC research has not only advanced our understanding of intestinal physiology but also revealed pathological roles of ISCs in diseases and facilitated the identification of potential targeted therapies. This review aims to comprehensively present the current state of ISC research. It elaborates on the multi-level regulatory mechanisms governing ISCs, including niche cells, signaling pathways, gut microbiota, extracellular matrix, and epigenetic regulation. It further summarizes the involvement of ISCs in colorectal cancer, inflammatory bowel disease, radiation-induced intestinal injury, and short bowel syndrome, as well as their application in organoid technology. Finally, this review highlights future directions, including dissecting how distinct cellular states and microenvironments dynamically regulate ISC function, and the integration of cutting-edge technologies like microfluidic organ-on-a-chip and gene editing technologies to accelerate the translation of basic discoveries into clinical practice, thereby providing a valuable reference for researchers in the field.

## 1. Introduction

The intestinal epithelium serves as a dynamic and critical barrier and undergoes complete renewal every 3–5 days. This remarkable regenerative capacity is driven by a highly regenerative stem cell population with the capacity to produce all differentiated cells [1]. Intestinal stem cells (ISCs), resident at the base of the intestinal crypts, give rise to transit-amplifying (TA) cells, which then differentiate into secretory cells and absorptive cells along the crypt–villus axis, ensuring continuous epithelial renewal and maintaining intestinal structural integrity and functional homeostasis [2]. ISCs are not only the driving force for the continuous renewal of intestinal epithelium but also the key performers in repairing intestinal injury. When intestinal tissue is damaged, ISCs can rapidly proliferate and replenish damaged intestinal epithelial cells (IECs), thereby restoring intestinal structural and functional homeostasis, which can be used as a therapeutic target for a variety of intestinal diseases [3].

**Figure 1:**
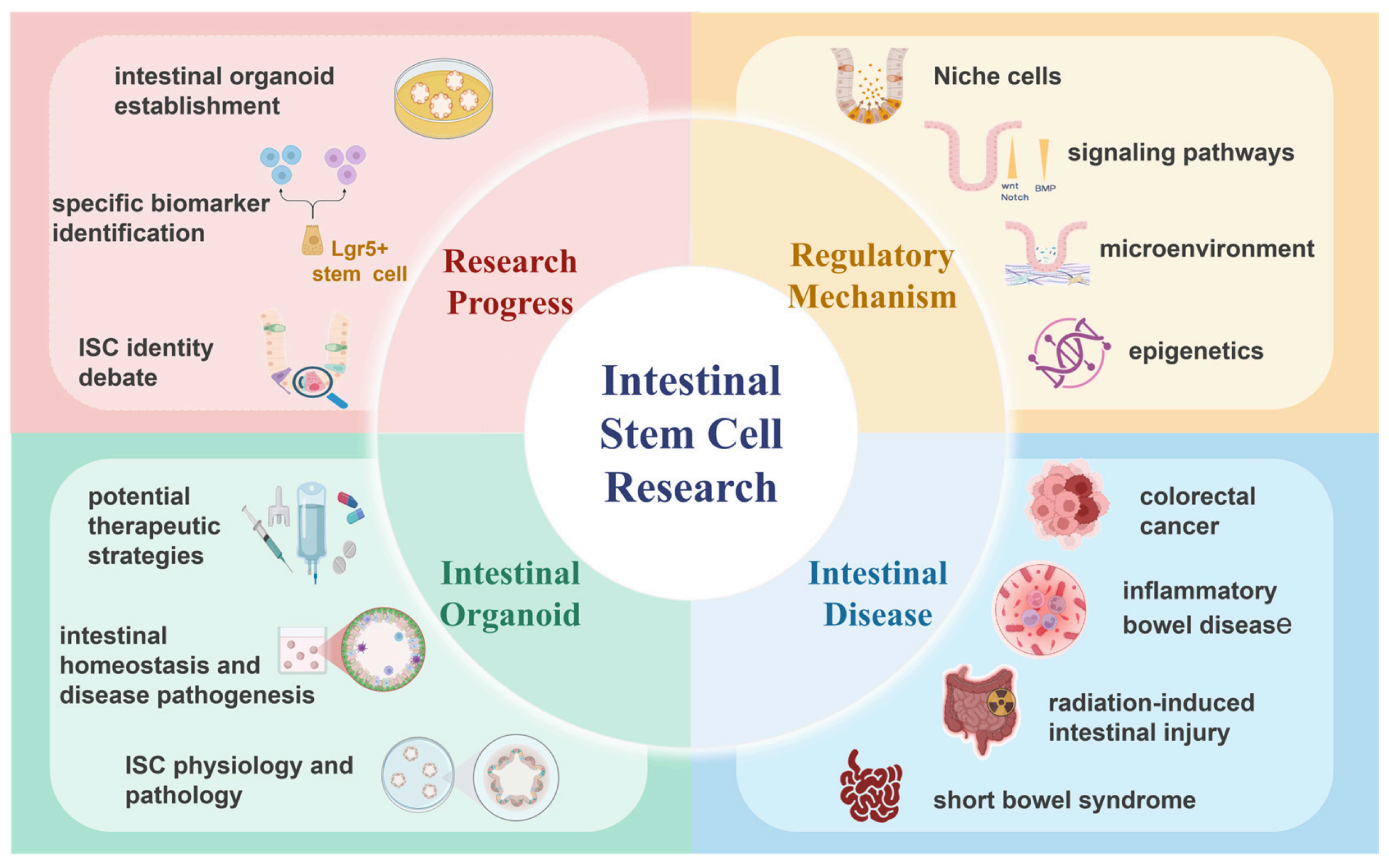
Graphic abstract. ISC: intestinal stem cell. Image created with BioRender.

Research on ISCs began gaining substantial momentum at the end of the 20th century. Since Lgr5 was identified as a marker of ISCs, ISC research has made an explosive breakthrough, establishing a robust foundation for the precise identification and isolation of ISCs and, in turn, has significantly clarified their roles in both physiological homeostasis and pathological processes [4]. In addition, the elucidation of the physiological functions of ISCs has greatly promoted the development of intestinal organoid technology, which has provided an important experimental paradigm for the construction of disease models, the accurate screening of therapeutic targets, and personalized drug sensitivity experiments [5,6]. Therefore, ISC research has become central to studies of intestinal biology, disease mechanisms, and precision medicine. However, few studies have systematically reviewed the current research progress and potential research hotspots of ISCs. Thus, this review aims to discuss the development and research progress of ISC research, hoping to provide a clear knowledge framework in this field. (Figure 1).

## 2. The Development of ISC Research

Intestinal stem cell (ISC) research has gone through several critical stages. In the 1970s, researchers Cheng and Leblond used the DLB-1 clone labeling technique to infer from morphological observations that the crypt basal columnar cells located at the bottom of the small intestinal crypts were ISCs [7]. Around the same time, Potten identified cells at the crypt “+4” position as ISCs. Due to a lack of direct evidence, neither idea was confirmed [8,9]. It was not until 2007 that the Hans Clevers group provided the first direct proof. Using lineage tracing, they showed that Lgr5+ crypt basal columnar cells can differentiate into all types of intestinal epithelial cells. Since then, Lgr5 has been considered a core marker of ISCs [10]. Some studies have reported that the “+4” population largely consists of early secretory progenitors derived from Lgr5+ ISCs, rather than a distinct stem cell population [11,12]. However, an alternative perspective suggests that “+4” cells act as quiescent stem cells. These cells, marked by Bmi1, remain quiescent under homeostatic conditions and do not contribute to regular epithelial renewal, but they can mediate ISC regeneration after injury [2].

As research advanced, our understanding of ISCs has become more comprehensive, and Lgr5+ ISCs are not the only cell type that maintains intestinal homeostasis. Using lineage tracing, researchers have found that prominin-1+ cells are located at the base of the small intestine, co-express Lgr5, and can also generate all lineages of IECs [13]. A unique cell type has been identified in the gut, known as revival stem cells (revSCs), which expand rapidly to restore intestinal homeostasis when Lgr5+ ISCs are eliminated [14]. In intestinal injury, differentiated daughter cells, such as tuft cells that serve as a reserve pool of ISCs, can regain ISC properties through dedifferentiation [15,16]. These results suggest that a specific stem cell pool is not the only central driver of intestinal epithelial regeneration. In a broader sense, stemness may represent a dynamic cellular state rather than a specific cell type. Instead, it is more likely to reflect a specific cell state.

Despite this dynamic and flexible view of stemness, the identification of specific markers for ISCs remains critically important. Such markers allow researchers to trace cell fate and dissect regulatory mechanisms even within a changing cellular landscape. Lgr5+ ISCs remain central to ISC research and are the most deeply studied stem cell type, serving as the primary marker that has enabled subsequent investigations into the regulatory mechanisms of ISCs [11].

The balance between self-renewal and differentiation of ISCs is precisely regulated by complex microenvironmental signals from niche cells such as mesenchymal cells and Paneth cells, which jointly modulate Wnt, BMP, Notch, and other signaling pathways. Understanding the physiological mechanisms of ISCs has not only deepened our knowledge of gut homeostasis but also paved the way for clarifying disease mechanisms and developing targeted therapies. Most studies have confirmed that the abnormal activation of the Wnt signaling pathway is closely related to the occurrence of colorectal cancer (CRC), and inhibiting the overactivation of the Wnt signaling pathway can effectively suppress CRC progression [17,18,19,20]. In chemotherapy-induced intestinal mucosal injury, ISC dysfunction is also a crucial reason for the failure of intestinal epithelial regeneration, and targeted regulation of ISC function has become a potential therapy. Thus, from the discovery of specific markers to the mapping of regulatory networks, and further to the clarification of pathological mechanisms and targeting strategies, ISC research is continuously promoting the transformation of basic research achievements into clinical practice.

In addition, given the high plasticity of ISCs, organoid technology has also progressed rapidly. In 2009, Hans Clevers’ team successfully constructed the first intestinal organoid with a crypt–villi three-dimensional structure by co-culturing single Lgr5+ ISCs with Matrigel and specific growth factors. This intestinal organoid showed high similarity to the human intestine in both structure and function, which provided a near-physiological in vitro model for subsequent mechanistic research and drug screening [21]. Since then, research focus has gradually shifted to clinical application, including the construction of patient-derived tumor organoid biobanks for drug sensitivity prediction, personalized treatment, and the exploration of regenerative medicine strategies based on organoid transplantation [22]. Research has also proposed that transplanting small intestinal organoids into the colon could generate a functional ‘small-intestinalized colon’, offering a new therapeutic approach for intestinal diseases [23,24,25].

Intestinal organoid technology is often integrated with other cutting-edge research methods. Among them, the microfluidic chip system derived from the combination of microfluidic technologies can not only achieve the dynamic culture of intestinal organoids, but also simulate the complex microenvironment of the intestine [26]. Thus, it can be used to dissect the interaction between different components of the microenvironment and ISCs [5]. Additionally, the integration of gene editing technology can regulate the expression level of specific genes in intestinal organoids to accurately simulate the disease process or carry out targeted therapy research [25]. Therefore, the deep integration of intestinal organoid technology and cutting-edge technology provides a powerful experimental platform for elucidating the pathological mechanisms of intestinal diseases and developing precise therapeutic strategies [27,28].

## 3. Regulatory Mechanisms of ISCs

**Figure 2:**
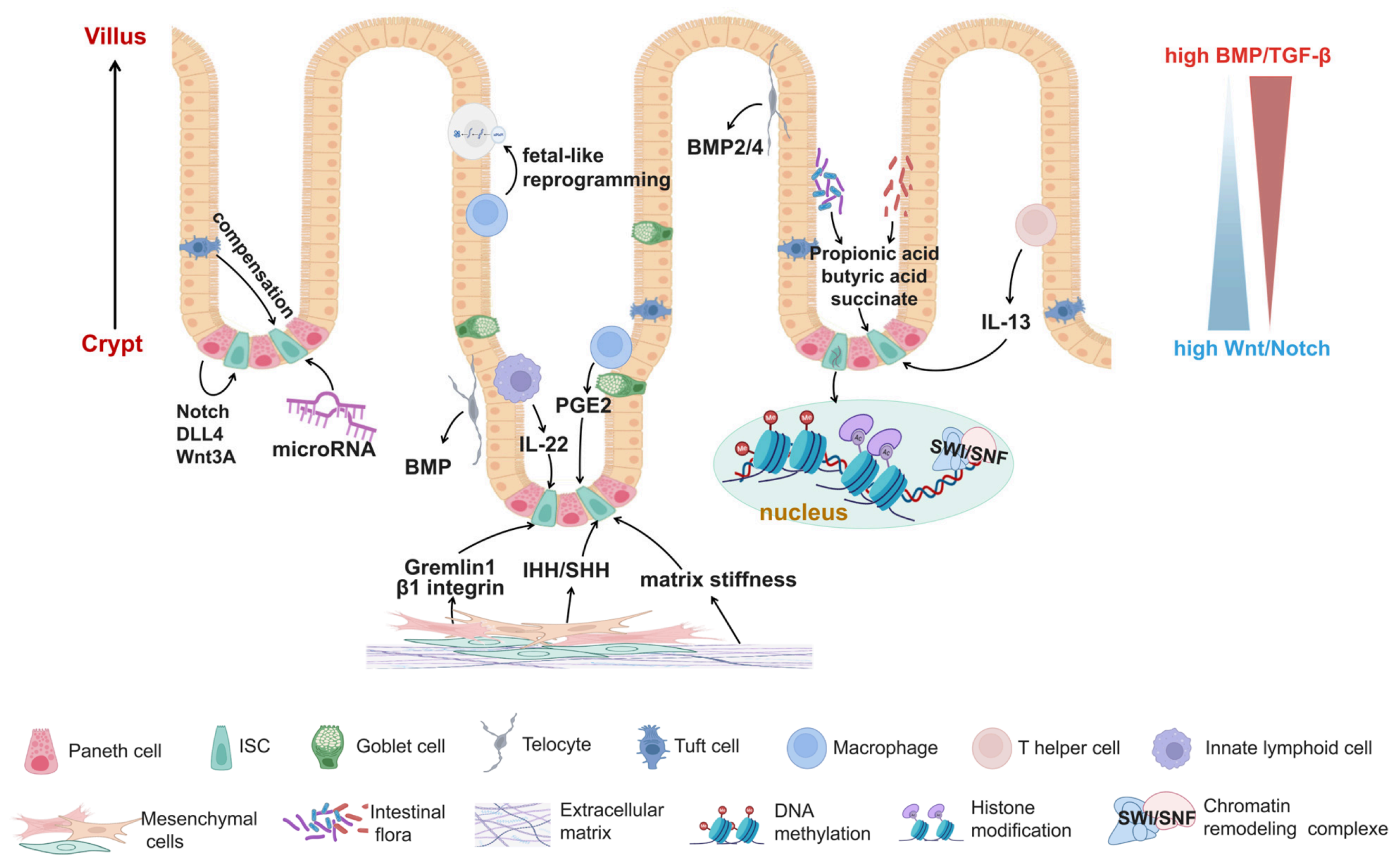
The regulatory mechanism of ISCs. This figure demonstrates the regulatory mechanism of the intestinal stem cell (ISC) function. The upper right depicts the gradient distribution of classical signaling pathways along the crypt–villus axis. ISCs reside at the base of the crypts, close to Paneth cells, and their activity is orchestrated by diverse factors. In the microenvironment, Paneth cells, extracellular matrix, mesenchymal cells, and telocytes modulate ISC function primarily through ligand secretion and subsequent pathway regulation. Immune cells mainly regulate ISC function via inflammatory cytokines, and macrophages can further regulate stemness through reprogramming. The intestinal microbiota regulates ISC function mainly via its metabolites. Epigenetics, including DNA methylation, histone modification, chromatin remodeling complexes, and microRNA, can directly modulate ISC activity. Furthermore, in the absence of ISCs, tuft cells can replenish the stem cell pool. These factors form a complex regulatory network governing ISC function. ISC: intestinal stem cell. Image created with BioRender.

ISC behavior is dynamically coordinated by a highly integrated niche environment. Rather than acting independently, signaling pathways, niche cells, microbial metabolites, and biomechanical cues collectively determine ISC fate decisions under both physiological and pathological conditions. These factors can precisely regulate ISC behavior through multi-level mechanisms [29]. In this review, we summarize the regulatory mechanisms of ISCs, with the signaling pathways involved presented in Table 1 and the contributions of niche cells, microenvironment, and epigenetic factors illustrated in Figure 2.

### 3.1. The Regulatory Mechanism of the Signaling Pathway on ISCs

#### 3.1.1. The Wnt Signaling Pathway

The Wnt signaling pathway is the core signaling pathway that regulates the activity of ISCs [30]. The distribution of signals along the crypt–villus axis shows a gradually decreasing trend, which ensures the high proliferative stemness of intestinal crypts [31,32]. Wnt ligands secreted by Paneth cells and stromal cells can bind to Frizzled receptors and their co-receptors LRP5/6 on the surface of ISCs, activating disheveled intracellular effectors and the Wnt pathway [33,34]. Subsequently, β-catenin translocates into the nucleus, where it binds to TCF/LEF family transcription factors, driving transcription of downstream target genes, including Lgr5, Ascl2, and c-Myc, and thereby promoting ISC expansion [35]. However, it is worth noting that excessive activation of Wnt signaling (such as APC gene mutations) promotes ISC hyperproliferation and induces colorectal cancer [36]. When Wnt is in a repressed state, the cytoplasmic destruction complex (consisting of Axin, colorectal adenoma polyps (APC), casein kinase 1(CK1), and glycogen synthase 3 (GSK3)) will phosphorylate β-catenin, leading to β-catenin degradation and impaired ISC activity [37,38].

#### 3.1.2. The Notch Signaling Pathway

The Notch signaling pathway regulates the differentiation fate of ISCs through direct interaction between adjacent cells [39]. This pathway relies on the binding of transmembrane Notch receptors (Notch1-Notch4) expressed on the surface of ISCs or their adjacent cells to ligands (DLL1/3/4, Jag1/2), triggering the release of the Notch intracellular domain (NICD) after cleavage of the receptor by proteases. NICD is then translocated into the nucleus and binds to the transcription factor CSL (also known as RBP-J) to form the NICD/CSL transcription complex, which upregulates the expression of target genes such as HES and Hey to regulate the differentiation of ISCs [40,41,42]. The Notch pathway plays a dual regulatory role in ISC homeostasis. Physiological activation of Notch signaling promotes ISC proliferation and maintains the differentiation direction of absorptive cell precursors [43]. Excessive activation leads to abnormal expansion of ISCs, whereas inhibition of Notch signaling drives ISC over-differentiation into secretory lineages, particularly goblet cells and Paneth cells, which drives intestinal dysfunction [44,45]. This fine-tuning mechanism is important for maintaining the balance between absorptive and secretory cell lineages in the intestine.

#### 3.1.3. The BMP Signaling Pathway

The BMP signaling pathway relies on the binding of transmembrane serine/threonine kinase receptors (BMPR1/2) expressed by IECs to the corresponding ligands (BMP2/4, etc.), triggering the activation of downstream Smad1/5/8 transcription factors after receptor phosphorylation [46]. Phosphorylated Smads are subsequently translocated into the nucleus to form a transcriptional complex with Smad4, which in turn upregulates the expression of differentiation-related target genes and indirectly inhibits Wnt pathway activity [47]. The BMP signaling pathway plays a gradient regulatory role in ISC homeostasis [48]. High concentrations of BMP antagonists (such as Noggin, Grem1) at the bottom of crypts balance the proliferation and self-renewal of ISCs by inhibiting BMP signaling. The increased BMP activity along the crypt–villi axis can antagonize Wnt signaling and drive cell differentiation toward the absorptive lineage [49,50]. Conversely, inhibiting the BMP signaling pathway can break the balance between cell proliferation and differentiation, which may lead to over-expansion of ISCs and abnormal crypt structure.

#### 3.1.4. The TGF-β Signaling Pathway

The TGF-β signaling pathway binds to activated ligands (TGF-β1/2/3) through transmembrane serine/threonine kinase receptors (TGFBR1/2), triggering receptor phosphorylation that activates downstream Smad2/3 transcription factors [51]. Phosphorylated Smad2/3 translocates into the nucleus and forms a transcriptional complex with Smad4, which upregulates the expression of cell cycle inhibitors (such as p21 and p15) and pro-differentiation/apoptosis-related target genes [52]. This pathway also plays a dual role. Under normal homeostasis, the TGF-β pathway can antagonize Wnt and inhibit excessive proliferation [53]. But when tissue damage or inflammation occurs, the TGF-β pathway is highly activated, and the regeneration-associated epithelial reprogramming is induced to remodel the microenvironment to support regenerative repair [54,55]. While excessive inhibition of the TGF-β pathway will release cycle constraints, triggering abnormal expansion of stem cells [56,57].

#### 3.1.5. The Hippo Signaling Pathway

The Hippo signaling pathway regulates ISC activity by phosphorylating the effector molecule YAP/TAZ through upstream kinase MST/LATS. When the upstream kinase MST1/2 activates LATS1/2, the phosphorylation modification of YAP/TAZ allows it to stay in the cytosol and be ubiquitinated for degradation, thus turning off the proliferative transcriptional program [58]. Conversely, when the upstream kinase MST/LATS cascade is inhibited, LATS1/2 fails to phosphorylate YAP/TAZ, allowing dephosphorylated YAP/TAZ to translocate into the nucleus and bind to TEAD transcription factors, upregulating CTGF, CYR61, and Wnt-promoting target genes [59,60]. Under normal circumstances, the intercellular connections between crypt cells are tight. The inhibitory signals from cell contact activate the Hippo pathway, and YAP/TAZ is confined to the cytoplasm to prevent excessive proliferation of ISCs and maintain intestinal homeostasis [61,62]. Once the intestinal tissue is damaged and the cell density decreases, the Hippo pathway becomes inactive. The downstream key effector proteins YAP/TAZ enter the nucleus to promote intestinal injury repair [63]. In addition, the Hippo pathway can also induce fetal-like transcriptional programs to support intestinal injury repair [64].

#### 3.1.6. The Hedgehog Signaling Pathway

The Hedgehog signaling pathway indirectly regulates ISC function through epithelial–mesenchymal paracrine interaction [65,66]. It relies on IHH/SHH ligand binding to the receptor Patched (Ptc) to unblock its inhibition of Smoothened (SMO), prompting Gli transcription factors into the nucleus to upregulate target gene expression [67]. In homeostasis, IHH acts as a negative feedback signal to limit the overactivity of the Wnt pathway in crypts and maintain homeostasis [68]. In injury, Hedgehog signaling can regulate the production of Upd2 in the daughter cells of ISCs to activate the JAK-STAT pathway to drive proliferation in Drosophila [69]. Whereas specific knockout of IHH could cause Wnt pathway-driven crypt hyperexpansion [66], and long-term inhibition of Hedgehog signaling disrupts the microenvironment, leading to dysfunction of the Wnt pathway and crypt disruption [66,70].

The regulation mechanism of signaling pathways on the activity of ISCs is complicated (Table 1). Along the intestinal crypt–villus axis, Wnt and Notch pathway signals show a decreasing gradient, with the highest concentration at the bottom of the crypt, jointly maintaining the self-renewal and stability of the ISC pool [71,72]. The Wnt pathway can promote the expression of Notch ligands and cooperatively guide the differentiation of daughter cells [73]. In contrast, the BMP pathway shows an increasing gradient with the TGF-β signaling, with the highest activity in the villus region [74]. The BMP pathway focuses on promoting IEC differentiation, while the TGF-β pathway regulates goblet cell differentiation and intestinal immune response [51,75,76]. The Wnt pathway inhibits the BMP pathway activity by inducing the expression of BMP antagonists at the crypts, and synergistically regulates ISC activity [77]. The Hedgehog pathway regulates the expression of BMP and Wnt ligands and indirectly participates in epithelial differentiation [78,79]. The Hippo pathway integrates BMP signaling and antagonizes Wnt output to limit crypt overproliferation under steady-state conditions, and cooperates with Wnt to promote regenerative repair under injury [80,81,82]. Recent research has discovered that chromatin rearrangement and the Hippo/TGF-β pathway jointly drive the generation of regenerative stem cells, which differentiate into new ISCs and repair damaged intestinal tissues [55].

Therefore, the functions of ISCs under physiological and pathological conditions are regulated by a complex network of multiple signaling pathways. However, whether there are differences in the synergy among the signaling pathways in these two different states, and the core mechanism leading to the differences, remains to be elucidated, which will be the key to further understanding the regulatory mechanism of ISCs.

### 3.2. The Regulatory Mechanism of Niche Cells on ISCs

Paneth cells, located at the base of crypts and closely adjacent to ISCs, are important niche cells of ISCs. They can secrete ligands such as Wnt3A, DLL4, and Notch to activate the Wnt and Notch pathways [83]. And it secretes antimicrobial peptides such as α-defensins and lysozyme to maintain the local microenvironment of crypts and prevent pathogens from invading, and affect neighboring ISC behavior by regulating local Β4 integrins [84]. Studies have shown that ablation of Paneth cells does not affect the ISC phenotype, suggesting that Paneth cell functions may be replaced by other cells [85]. In contrast, research has discovered that Paneth cells can be transdifferentiated into ISCs to participate in repairing intestinal injury, as it is directly adjacent to ISCs and can provide essential short-range niche signals to adjacent ISCs [86].

Mesenchymal cells fine-tune ISCs through the cooperation and antagonism of multiple subsets. These cells are spatially distributed along the crypt–villus axis and the small intestine–colon axis, and have plasticity in the injury state, which can dynamically regulate ISCs. Trophocytes at the base of crypts (CD81^+^ PDGFRA (lo)) have been reported to secrete the BMP inhibitor Gremlin1, while PDGFRA(hi) telocytes at the base of the villi provide BMP signaling [48]. Lgr5^+^ telocytes at the tip of the villi can secrete Wnt5A, Rspo3, BMP2, and BMP4, which together regulate the terminal differentiation of IECs [87]. Some mesenchymal cell functions are redundant, and the depletion of a single subpopulation can be compensated for by other subpopulations, but the complex network formed by the whole ensures the precise control of ISCs in homeostasis and regeneration. In addition, mesenchymal cells interact with epithelial cells. Mesenchymal cells with high expression of Pdgfrα can activate epithelial Wnt signaling, promote Shh Expression, and regulate villous morphogenesis [88]. However, the mechanism of how the crosstalk between mesenchymal cells and IECs regulates ISCs has not been fully elucidated [89].

Intestinal immune cells can also regulate ISC function. Innate lymphoid cells (ILC) located around crypts can secrete IL-22, bind to IL-22 receptors on ISCs, and activate the STAT3 pathway to promote ISC proliferation [90,91]. ILC2 can promote the differentiation of tuft and goblet cells by secreting IL-13 [92]. T helper cells can regulate the differentiation function of Lgr5^+^ ISCs by controlling the release of inflammatory factors [93]. In addition to releasing inflammatory factors, immune cells can also regulate the function of ISCs in other ways. Macrophages can migrate around ISCs, promote fetal-like reprogramming of IECs, and induce cell regeneration to repair the intestinal injury [94]. Thus, immune cells can form a dynamic interaction network with ISCs under physiological and pathological conditions, serving as an important regulatory force in maintaining intestinal epithelial homeostasis.

### 3.3. The Regulatory Mechanism of the Microenvironment on ISCs

#### 3.3.1. The Intestinal Flora and Metabolites

As a complex microbial community, the gut microbiota plays a pivotal role in modulating ISC activity. Early-life microbiota induces Erdr1 expression in ISCs to promote intestinal development and repair [95]. Beneficial bacteria such as *Akkermansia*, *Duboisella*, and *Lactobacillus* enhance epithelial integrity [96]. Actually, microbial heterogeneity exerts different effects [97]. It has been reported that obese Meishan pigs exhibit deeper crypts and stronger ISC proliferation, associated with a higher abundance of active *Lactobacillus*, whereas tilimycin-producing (til+) *Klebsiella* produces the genotoxin tilimycin, driving colonic stem cell mutations and disease [98]. Additionally, given the compositional and functional complexity of the microbiota, its regulatory influence on ISCs remains unclear. Most research has focused on single bacterial species, but the microbiota’s physiological impact depends on its network-level structure, and probiotic supplementation shows limited efficacy [99,100]. Thus, investigating multi-strain microbial network synergies is essential for future therapeutic strategies.

Microbiota metabolites also affect ISCs. *Akkermansia* is reported to promote Paneth and goblet cell differentiation by enhancing propionic acid metabolism [101,102]. Butyrate supports healthy intestinal cell renewal while inhibiting CRC stem cell proliferation via HDAC and Wnt pathways [103]. However, some metabolites inhibit ISC regeneration. A. Radioresistance has been shown to produce indole-3-acetic acid (IAA), which activates Ahr and suppresses Wnt-Ephb2 signaling [104]. *Enterococcus faecalis*-derived tyrosine impairs epithelial regeneration and exacerbates colitis [105]. Thus, regulation of microbiota metabolites is complex, and how distinct metabolic signals converge to modulate ISC function remains poorly understood. A key challenge lies in achieving precise modulation of microbial metabolic patterns to control ISC function.

Additionally, the microbiota indirectly shapes ISC function via interactions with other host systems. Microbiota-immune crosstalk is particularly notable. It has been reported that inulin promotes γδT cell-derived IL-22 secretion to restore colonic homeostasis [106]. *Lactobacillus* and its metabolite IAA stimulate IL-22 production from lamina propria lymphocytes, accelerating ISC proliferation and mucosal repair [107]. Furthermore, gut microbiota-enteric nervous system crosstalk has also been shown to regulate ISC function. Valeric acid has been reported to enhance 5-HT-associated signaling, which drives macrophage PGE2 production, enhancing ISC proliferation [108]. Despite these insights into multi-system interactions, the precise cooperative mechanisms regulating ISCs require further investigation.

#### 3.3.2. The Extracellular Matrix

The extracellular matrix (ECM) is a three-dimensional structure composed of various proteins secreted by epithelial cells and stromal cells. The ECM proteins, such as fibronectin and collagen, provide positioning and anchoring support for the ISCs, and also ensure that signaling molecules are efficiently enriched locally in the crypts [109]. By binding to integrins or discoidin domain receptors on ISCs, the ECM activates intracellular signaling pathways that regulate ISC regeneration and renewal [110]. For example, β1 integrin in the ECM mediates Hedgehog signaling to regulate ISC proliferation [70], and heparan sulfate proteoglycans (HSPGs) can promote ISC regeneration by modulating the Wnt/β-catenin pathway [111].

Additionally, the stiffness of the ECM is also an important factor regulating the renewal and regeneration of ISCs. Matrix stiffness ensures the optimal proliferative state of ISCs in vitro [112]. The Hippo/YAP pathway is the main effector pathway for sensing changes in matrix stiffness and mediating the regulatory effects of the ECM. Matrix stiffness can promote YAP nuclear translocation, initiating the ISC proliferation and differentiation program [113]. Naturally, the Hippo pathway is not the only responsive pathway. A recent study has shown that changes in matrix stiffness can regulate the Notch pathway by stimulating Piezo ion channels to drive the differentiation of secretory cell lineages [114]. Therefore, the ECM and cell-signaling pathways together form a highly coordinated functional network that ultimately achieves precise regulation of ISC regeneration.

### 3.4. The Regulatory Mechanism of Epigenetics on ISCs

Epigenetics is a biological process that regulates gene activity or phenotype without altering the DNA sequence, thereby influencing tissue function. The main mechanisms include DNA methylation, histone modification, chromatin remodeling, and non-coding RNA [115,116]. DNA methylation is one of the most extensively studied mechanisms in DNA. It involves the covalent addition of a methyl group to the 5-carbon position of cytosine residues, primarily at CpG dinucleotide sites, catalyzed by DNA methyltransferases (DNMTs), thereby regulating cellular functions [117]. Studies have shown that DNA methylation dynamically determines the stemness maintenance and differentiation direction of ISCs [118,119]. Inflammation-induced accumulation of metabolites, such as succinate, can limit the regenerative potential of ISCs through persistent DNA methylation reprogramming [120].

In addition, histone modification is also an important link in the regulation of ISCs. Histones regulate gene expression mainly via N-terminal post-translational modifications, including acetylation, methylation, phosphorylation, and ubiquitination [121]. H3K4me3/27AC is enriched in the promoter regions of stem cell-related genes to promote transcription. G9a-mediated H3K9me2 epigenetically silences cell cycle negative regulatory genes to drive repair [122].

The core function of chromatin remodeling is to alter the compactness of chromatin, switching it from a closed state to an open state, thereby allowing regulatory proteins to access and bind to specific DNA sequences. Among the chromatin remodelers, the SWI/SNF family is a well-characterized complex that exhibits prominent functions [123]. Chromatin remodeling complexes, such as SWI/SNF, regulate ISC accessibility to Wnt signaling [124]. Crypt cell-specific chromatin remodeling converges on the TGF-β and Hippo signaling pathways, directly inducing functional revSCs and thereby initiating the intestinal regenerative program [55].

Non-coding RNAs (ncRNAs), such as circular RNAs and microRNAs (miRNAs), serve as indispensable regulatory factors in the process of epigenetic transcriptional regulation. Among circular RNAs, CircEZH2_005 promotes Lgr5+ ISC proliferation and attenuates I/R-induced intestinal mucosal injury via direct binding to RNA-binding proteins [125]. While circBtnl1 inhibits ISC self-renewal through transcriptional regulation of Sox9 [126]. Additionally, miR-29a/b promotes ISC regeneration by inducing metabolic reprogramming [127], and miR-958 regulates ISC numbers by targeting BMP signaling [128]. Another study reported that miR-200a-3p, miR-101-3p, and miR-34a-3p could suppress the stemness of intestinal organoids [129]. In the context of radiation-induced intestinal injury (RIII), miR-34a has also been identified as a key mediator of intestinal epithelial cell death, further highlighting the involvement of miR-34a family members in ISC regulation under both physiological and pathological conditions [130]. These results indicate that ncRNAs exert complex regulatory functions in modulating ISC function.

## 4. Research Status of ISCs in Intestinal Diseases

**Figure 3:**
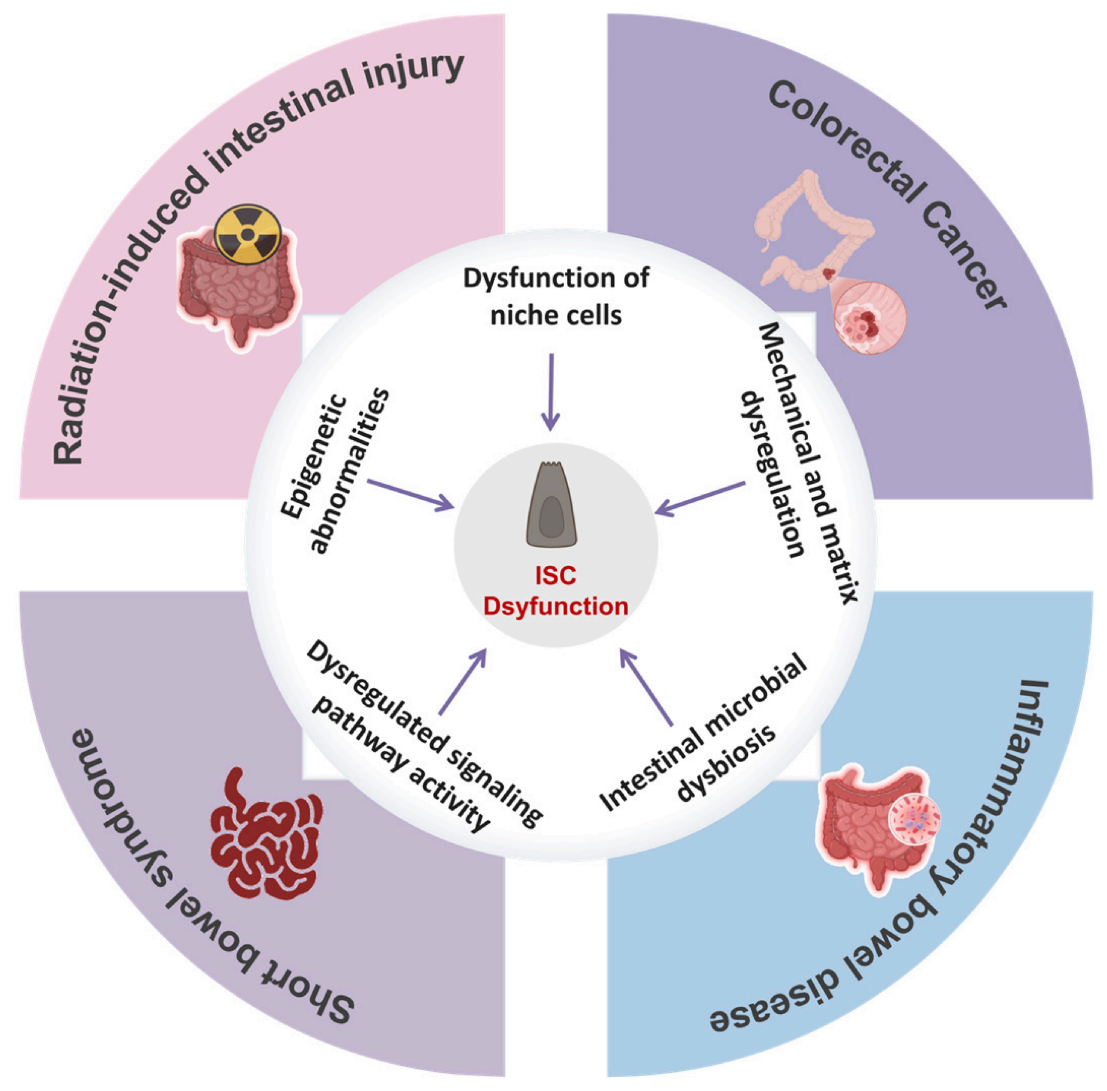
Common intestinal diseases arising from ISC dysfunction. The central panel illustrates key pathological factors that compromise ISC function, including dysfunction of niche cells, mechanical and matrix dysregulation, intestinal microbial dysbiosis, dysregulated signaling pathway activity, and epigenetic abnormalities. The surrounding panels summarize the common intestinal diseases resulting from ISC dysfunction, including colorectal cancer, inflammatory bowel disease, radiation-induced intestinal injury, and short bowel syndrome. ISC: intestinal stem cell. Image created with BioRender.

Disruption of ISC regulatory networks, including dysfunction of niche cells, mechanical and matrix dysregulation, intestinal microbial dysbiosis, dysregulated signaling pathway activity, and epigenetic abnormalities, can cause intestinal diseases. Among them, colorectal cancer (CRC), inflammatory bowel disease (IBD), short bowel syndrome (SBS), and radiation-induced intestinal injury (RIII) are the most widely reported types of intestinal diseases in the field of ISC research (Figure 3).

### 4.1. Colorectal Cancer

CRC is a malignant tumor that originates in the mucosa of the colon or rectum. It is the third most common cancer worldwide, with a high incidence and mortality rate [131]. In recent years, driven by shifts in dietary patterns, increasingly sedentary behaviors, and potential psychosocial stress factors, the incidence of CRC among young adults has risen, posing an increasingly serious public health challenge [132]. Current research indicates that the occurrence of CRC is predominantly driven by the accumulation of genetic mutations that lead to the uncontrolled proliferation of ISCs [133]. Aberrant self-renewal and plasticity of ISCs are considered major drivers of CRC initiation, progression, and metastasis. CRC pathogenesis is commonly initiated by APC mutations in ISCs, followed by additional alterations in KRAS, TP53, and SMAD4 during adenoma-carcinoma progression. These mutations collectively drive abnormal ISC proliferation [134]. Additionally, differentiated cells can regain stem-like properties through processes involving dysregulated expression of stem cell regulators such as SOX9, which is also recognized as a significant driver of colorectal carcinogenesis [135].

In recent years, the intestinal microenvironment has gained increasing attention due to its role in CRC development. Studies have shown that genetically mutated crypt ISCs can accelerate tumor metastasis through paracrine mechanisms that affect adjacent crypt tissues [136]. Dysregulation of enteric nervous system-derived 5-hydroxytryptamine (5-HT) signaling has been implicated in promoting CRC progression [137]. More recently, Ptgs2-positive fibroblasts within the mesenchymal niche were found to modulate intestinal tumor stem cell behavior via the PGE2-PTGER4-YAP signaling axis in a paracrine manner [138]. Despite these insights, the pathogenesis of CRC remains complex, and a comprehensive understanding of how various microenvironmental factors coordinately regulate ISC function has yet to be fully elucidated.

With a deeper understanding of the ISC pathological mechanisms in CRC, potential therapeutic targets have become a research hotspot. As the core regulatory pathway of ISCs, inhibition of the Wnt pathway represents an important breakthrough in CRC treatment [139,140]. However, how to regulate the Wnt pathway in cancer stem cells without interfering with normal ISCs is an urgent problem that needs to be solved in future research. On the other hand, in clinical treatment, the emergence of drug resistance severely limits the treatment effect and prognosis improvement of CRC patients [141]. The regeneration-associated stem cell state has been identified as a pivotal factor leading to drug resistance in treating CRC [142]. However, the specific mechanism of regeneration-associated stem-like cells in drug resistance has not been systematically elucidated. Thus, how to regulate the functional state of these cells to reduce the occurrence of drug resistance is an urgent issue that needs to be solved in CRC treatment.

In summary, owing to the high heterogeneity and plasticity of CRC stem cells, current clinical interventions are still very limited. Therefore, comprehensive elucidation of the pathogenesis of CRC and exploration of effective therapeutic targets are still the core issues that need to be addressed in the future.

### 4.2. Inflammatory Bowel Disease

IBD is a group of chronic, relapsing intestinal disorders characterized by persistent intestinal inflammation, with abdominal pain, diarrhea, and hematochezia as its predominant clinical manifestations [143]. It includes ulcerative colitis (UC) and Crohn’s disease (CD) [144]. The global incidence of IBD has been increasing, posing a substantial burden on patients’ physical and mental health [145]. The etiology of IBD is multifactorial. Accumulating evidence suggests that genetic susceptibility, gut microbiota dysbiosis, epithelial barrier dysfunction, and aberrant immune responses collectively contribute to IBD pathogenesis [146,147]. Substantial evidence supports the notion that dysregulated intestinal inflammatory responses are key elements in its pathogenesis [148]. However, the existing clinical strategies targeting intestinal immune abnormalities remain limited. In recent years, growing evidence has established intestinal epithelial injury as a hallmark of IBD [149]. Consequently, investigating the pathogenesis of IBD and developing potential therapeutic approaches from the perspective of ISCs has emerged as a prominent research focus [150,151,152].

Studies have indicated that enhancing Wnt signaling and modulating mitochondrial metabolic patterns in ISCs can stimulate their regeneration, thereby ameliorating the symptoms of IBD [32,153,154]. However, few ISC-targeting therapeutics have been developed for clinical use in IBD. To further clarify the pathogenic role of ISCs in IBD, a recent study found that deletion of the SETDB1 gene in ISCs triggers irreversible stem cell-dependent apoptosis and induces severe intestinal inflammation [155]. Through integrative analysis of single-cell datasets from healthy and diseased intestines, a distinct cell population termed INFLAREs has been identified in patients with IBD. These cells are suggested to arise from ISC-associated regenerative programs, exhibit both stem-like properties and immunoreactivity, and represent a potential therapeutic target [156]. In addition, MG53 (also known as TRIM72) has been shown to promote asymmetric division of ISCs by activating PPARα signaling and driving ISC differentiation toward secretory lineages, which may support the restoration of intestinal epithelial homeostasis in IBD [157].

In summary, while current research has broadly expanded our understanding of ISCs in IBD pathogenesis, the functional connections among these mechanisms remain poorly understood. In particular, it remains unclear whether a central molecular node exists that integrates these disparate pathological processes. Therefore, future studies aimed at identifying more precise therapeutic targets should focus on elucidating these underlying mechanisms.

In addition, dysregulated intestinal immune responses are hallmarks of IBD pathology and are prone to forming a vicious circle of disorders with intestinal epithelial barrier dysfunction [149]. Therefore, recent studies have emphasized the importance of simultaneous regulation of the intestinal immune response and intestinal epithelial barrier function [158,159,160]. Recent studies have demonstrated that IL-17A specifically upregulates ATOH1 expression in ISCs, driving ISC differentiation into secretory lineage cells, a process implicated in intestinal epithelial repair. Furthermore, inhibition of TMEM219 signaling in ISCs suppresses Caspase-8-mediated inflammatory responses, ameliorates intestinal inflammation, and facilitates the regeneration of the intestinal mucosa [161]. To achieve precise treatment, a new drug delivery system has been developed to achieve dual regulation of immune modulation and intestinal barrier repair. An intestinal anti-inflammatory microsphere vehicle has been developed, and it can precisely target sites of colonic inflammation and responsively release MXene and L-arginine to regulate immune homeostasis, which indirectly remodels the ISC niche and promotes intestinal epithelial repair [162].

Thus, the current study emphasizes the synchronous and targeted modulation of both ISCs and intestinal immune response, underscoring the value of multi-target therapeutic strategies. However, the etiology of IBD is complicated, and the core pathological relationship between intestinal epithelial barrier disruption and the intestinal immune-inflammatory response remains unclear. Future research should further investigate the interactions between ISCs and immune cells to deepen our understanding of IBD pathogenesis and advance the development of multitargeted and precise treatment strategies.

### 4.3. Radiation-Induced Intestinal Injury

RIII is also an important disease type in the research on the pathological mechanism and potential treatment strategies mediated by ISCs [163,164]. RIII is one of the most common complications of radiotherapy [165]. Clinically, RIII is often characterized by diarrhea, abdominal pain, bloody stool, and intestinal obstruction, and may even progress to chronic fibrotic changes in the later stages, which seriously affect the prognosis of patients [166]. However, the current clinical treatment methods are relatively scarce and have limited efficacy, making RIII a major clinical challenge that urgently needs to be overcome in the field of radiotherapy [167].

The pathogenesis of RIII is highly complex, and the intestinal barrier damage is the core pathological mechanism of RIII. The intestinal barrier primarily consists of the mechanical, mucosal, and microbial barriers [168]. Irradiation damages intestinal villi, causes the loss of IECs, and leads to mechanical barrier abnormalities and increased intestinal permeability. Meanwhile, the mucosal barrier, mainly composed of goblet cells and their secreted mucins, is compromised by radiation-induced goblet cell loss, which weakens the defense against pathogens and exacerbates intestinal inflammation [169]. Furthermore, irradiation disrupts the structure and function of the intestinal microbiota, damaging the microbial barrier and promoting the overgrowth of pathogenic bacteria [170]. Therefore, restoring the structure and function of the intestinal epithelial barrier is crucial for alleviating RIII intestinal symptoms.

Irradiation can directly damage ISC, which is recognized as a critical driver factor in intestinal barrier damage [171]. Research has employed single-cell sequencing to reveal the dynamic patterns and roles of the three ISC subtypes in RIII, including Lgr5+ ISC, +4 reserve stem cells (RSCs), and a cluster of Clu+ revival stem cells (revSCs), suggesting a complex interconversion pattern among them in response to intestinal injury [172]. Mechanistically, radiation-induced damage triggers the secretion of interleukin-33 (IL-33) from ISCs. IL-33 subsequently acts on adjacent Paneth cells, stimulating the release of epidermal growth factor (EGF) and other niche signals, which in turn drive intestinal epithelial regeneration and promote restoration of the mucosal barrier [173]. Given the central role of ISCs in intestinal repair and regeneration, modulating key signaling pathways, such as Wnt, BMP, and Notch pathways, to regulate ISC activity represents a promising therapeutic strategy for ameliorating RIII [164,174]. Notably, activating the Wnt/β-catenin pathway not only promotes ISC proliferation and differentiation but also facilitates goblet cell maturation and mucin secretion, thereby repairing the intestinal barrier [175].

In addition, regulating intestinal microbiota balance is also an effective therapeutic approach for RIII. Certain beneficial bacteria have been identified as potent activators of ISC regeneration. Limosilactobacillus reuteri and Lactobacillaceae can activate the Wnt/β-catenin pathway to drive ISC-mediated regeneration, accelerating intestinal barrier repair and enhancing intestinal defense [176]. Moreover, intestinal microbiota-derived metabolites play a critical regulatory role. Faecalibaculum rodentium-derived butyrate has been shown to maintain intestinal barrier integrity and exert radioprotective effects [177]. Microbe-derived I3A could regulate probiotic abundance and promote the proliferation and differentiation of ISCs to facilitate intestinal barrier repair [166]. In addition, secondary bile acids activate the TGR5 receptor, which subsequently activates Wnt signaling via the cAMP-PKA cascade, thus promoting ISC regeneration [178].

However, the role of the gut microbiota in RIII has not been fully elucidated. The same species may exert opposite effects. Research showed that RIII-induced intestinal barrier dysfunction causes the overgrowth of *Akkermansia muciniphila*, which leads to decreased ISC density and suppressed goblet cell differentiation, thus further damaging the integrity of the intestinal epithelial barrier [179,180]. In contrast, other studies have reported that *Akkermansia* can activate the Wnt/β-catenin signaling pathway to promote ISC proliferation and secrete propionate to exert radioprotective effects [181,182]. This inconsistency in results may be related to irradiation intensity, disease stage, or the intestinal microenvironment. Therefore, further studies are needed to deeply investigate the specific pathological role of *Akkermansia muciniphila* in the pathogenesis of RIII [180]. Despite this complexity, it is clear that precisely modulating intestinal microbiota composition to facilitate ISC regeneration is a promising therapeutic strategy for repairing the intestinal barrier of RIII [183].

### 4.4. Short Bowel Syndrome

SBS is characterized by nutrient malabsorption due to shortened intestinal length, which may progress to intestinal failure in severe cases [184]. Studies have found that in the early adaptive response of intestinal resection, intestinal crypts are deepened, villi are elevated, and the function of nutrient absorption is enhanced, indicating that the regenerative activity of ISCs is activated [163]. However, another study in the intestine of non-fed SBS infants found that the proliferative response in the intestine was not apparent, even though ISC-related gene expression was upregulated, suggesting that the intestinal adaptive response of SBS not only requires the participation of ISC-related genes, but also requires physical stimulation and the regulation of the intestinal microenvironment [185]. Evidence indicates that targeting myofibroblasts in the mesenchymal niche can enhance the proliferative capacity of ISCs and improve the adaptive responses of the intestinal epithelium [163]. Furthermore, intestinal organoid transplantation has emerged as a potential therapeutic approach for SBS [186]. Recent advances in tissue-engineered small-intestine systems have substantially accelerated the clinical translation of engineered intestinal grafts. The ongoing development of functional tissue-engineered intestine is expected to open new avenues for curing SBS in the future [187].

Altogether, the dysfunction of ISCs is the core link in the occurrence and development of a variety of intestinal diseases. At present, the research of ISCs has gradually expanded from the biological mechanisms of CRC to non-malignant diseases such as IBD, and the research focus has also turned to epithelial regeneration in the complicated microenvironment and the interaction between ISCs and different components in the intestinal microenvironment [113,188]. This paradigm shift holds significant promise for deepening our understanding of disease pathogenesis and driving the development of novel therapeutic strategies [189,190]. However, at present, the role of ISCs in the pathogenesis of these diseases is still in the initial stages of exploration, and whether ISCs could be an effective target for regulating various intestinal diseases still needs to be explored in depth.

## 5. Development of Organoid Technology in ISC Research

Since the first successful culture of intestinal organoids in 2009, ISC research has reached a new stage. Compared with the traditional two-dimensional cell model, the intestinal organoid model has a three-dimensional structure and can better simulate the complex structure of human tissues, which not only significantly reduces the sacrifice of experimental animals but also deepens our understanding of how individual single stem cells shape the tissue structure [191]. The intestinal organoid model has evolved from an initial lumen-like structure to a monolayer format, providing a reliable platform for the study of ISC regeneration mechanisms and the screening of targeted medicine. Functional epithelial monolayers generated using refined techniques closely recapitulate the structural and physiological characteristics of the human intestine [192]. These systems are more capable of simulating physicochemical stimuli and can be integrated into co-culture setups, serving as critical tools for studying epithelial crosstalk with the enteric nervous system, immune components, and other relevant factors. Consequently, the intestinal organoid model narrows the structural and functional gaps between experimental animals and humans.

### 5.1. Intestinal Organoid Technology in Intestinal Physiology and Pathology

Intestinal organoids have facilitated the elucidation of the regulatory pathways in ISCs, providing a direct experimental platform for studying their physiological functions and exploring disease mechanisms [193]. Previous studies using intestinal organoid models have confirmed the involvement of multiple signaling pathways in the network regulation of ISC physiology and further revealed how these ISCs contribute to the formation of intricate crypt–villus architecture [83,194]. In addition, studies on the supportive roles of peripheral tissues such as the enteric nervous system, lymphatic system, and tuft cells in ISC regeneration have systematically elucidated the regulatory role of the intestinal microenvironment on the physiological functions of ISCs [1,195,196]. By culturing ISC-derived enteroids and employing transcriptomic sequencing to compare the morphological and physiological differences between infant and adult intestinal tissues, research not only elucidated the developmental physiology of the intestine but also paved the way for investigating the pathological mechanisms of age-specific diseases [197].

Intestinal organoids have also been widely used in the study of disease mechanisms and the exploration of therapeutic strategies. Human norovirus (HuNoV), a major causative agent of acute gastroenteritis, has long been refractory to conventional in vitro culture, which has significantly hindered related research. By establishing human intestinal organoid models, researchers have not only overcome this long-standing limitation but also identified candidate antiviral agents against HuNoV, thus breaking through the bottleneck in the clinical management of such intestinal infectious diseases [198,199]. Moreover, researchers have successfully achieved gene correction in intestinal organoids derived from patients with cystic fibrosis by combining intestinal organoid culture with CRISPR/Cas9-mediated genome editing. This finding demonstrates a promising therapeutic strategy for the treatment of inherited intestinal disorders [200]. Furthermore, a study that used a patient-derived cancer organoid biobank screened over 500 bispecific antibodies, among which MCLA-158 was found to effectively inhibit the growth of CRC organoids and prevent metastasis [201]. This research has significantly facilitated the exploration of potential therapeutic strategies for CRC treatment.

Apart from this, intestinal organ transplantation has also received considerable attention in the field of intestinal disease research. Research has shown that intestinal organoid transplantation could promote the regeneration of colonic epithelium in mice with refractory ulcerative colitis [24]. The intestinal organoid model has also shown promise in regenerative medicine. Research has demonstrated that transplantation of human ileal organoids into rats enables the formation of a “small intestinalized colon” (SIC). Remarkably, the SIC develops mature villi, vascularized stroma, and lymphatic structures, and markedly ameliorates intestinal failure in SBS rats. This finding provides a new therapeutic paradigm for SBS and contributes to the ongoing advancement of organoid-based regenerative medicine [6]. Thus, intestinal organoid models provide a highly controllable and efficient platform for mechanistic validation and pharmacological investigations.

Furthermore, a growing body of research indicates that intestinal organoids are implicated not only in intestinal disorders but also contribute to the development of extra-intestinal pathologies. Studies utilizing intestinal organoids have confirmed that activation of the Abelson tyrosine-protein kinase 1 (ABL1)-YAP signaling axis in ISCs can induce hepatic steatosis [202]. Furthermore, using an organoid culture system, researchers have systematically discovered the pathogenesis of intestinal failure-associated liver disease, a severe complication of short bowel syndrome (SBS), and have pinpointed potential therapeutic strategies [203]. These studies highlight ISC as a critical regulatory hub, uncovering the interactive mechanisms between different organs during disease progression. Such insights not only deepen our understanding of the holistic pathological network of diseases but also provide new directions for exploring therapeutic targets.

### 5.2. Convergence of Intestinal Organoid Technology and Advanced Technologies

The intestinal organoid models at present are still primarily based on self-assembled three-dimensional cellular structures but lack essential auxiliary tissue components such as blood vessels or lymphoid tissue [204]. Notably, organoid co-culture technology provides an effective platform to narrow the limitations. Co-culturing intestinal organoids with different intestinal components helps to explore the interaction between different components and ISCs or intestinal epithelium, thereby deepening our understanding of intestinal physiology. Co-culturing intestinal organoids with immune cells has revealed that type 1 innate lymphocytes can drive intestinal regeneration, and also confirmed the key role of IL-22 in promoting epithelial regeneration [205].

Additionally, co-culture technology is also important to explore intestinal disease mechanisms and develop therapeutic strategies. Research co-culturing intestinal organoids with fibroblasts and B cells has identified that B cells are a key factor affecting mucosal repair in IBD, which offered insights for improving IBD treatment [206]. Another study co-cultured primary tumor organoids from CRC surgical samples expressing different levels of carcinoembryonic antigen (CEA) with T lymphocytes, and showed that the T cell killing activity of CEA-CD3 T cell bispecific monoclonal antibody was closely related to CEA expression level [207]. This result can help to identify the CRC patients most likely to benefit from this medicine and support more precise treatment.

In recent years, the emerging microfluidic organ-on-a-chip technology has provided an approach to more accurately recreate the complex intestinal microenvironment and simulate fluid shear stress [5]. This platform can be used to study disease mechanisms and develop precise treatments. Currently, microfluidic organ chips are widely used in studies of microbial-epithelial interactions. By precisely controlling key parameters such as oxygen gradients and growth factors, this technology has effectively promoted research on intestinal microecology [208]. Research has used the chip technology to explore the influence of intestinal immune cells on intestinal function, and put forward the potential possibility of mucosal vaccination, which provides a new idea for the treatment of intestinal immune-related diseases [209].

To more precisely simulate the in vivo intestinal microenvironment and enrich the cellular diversity of organoids, a study has integrated organoid technology with organ-on-a-chip technology to construct a novel human organoid model named “mini-colon”. This model not only significantly extends the tissue culture period but also recapitulates the maturation status and proliferation-differentiation patterns of cells in vivo, thereby providing a more reliable platform for studying intestinal physiological and pathological mechanisms [210]. Currently, research has focused on developing “mini-colon” models derived from CRC patients to study the complexity of the tumor microenvironment, drug toxicity, and drug resistance. This has further enhanced our understanding of the occurrence and development of CRC [211,212]. In addition, the “mini-colon” model has been applied to evaluate the gastrointestinal toxicity of candidate drugs, which significantly facilitates the development of safer and more effective drugs [213].

Combining gene editing technology with intestinal organoids also offers a new research paradigm, as it can accurately regulate molecular balance and cellular function. Precise single-gene editing in intestinal organoids helps clarify how different signaling pathways contribute to CRC, providing a detailed explanation of CRC pathogenesis [214]. Beyond disease mechanisms research, the combination of intestinal organoids and gene editing technology also plays an important role in exploring precision treatments for intestinal diseases. In a study, the CRISPR-Cas9 technology was used to knock down the RNF43 gene fragment in intestinal organoids, which proved that RNF43 could activate Wnt signaling mutations during tumorigenesis and promote tumor growth [215]. This result provided more possibilities for targeted therapy of intestinal tumors. In another study, ISCs derived from patients with cystic fibrosis (CF) were cultured into organoids with gene editing technology to precisely repair the CFTR gene, thereby restoring intestinal organoid function. This demonstrates the great potential of personalized therapy for monogenic diseases [200].

The synergistic integration of multi-omics and intestinal organoid technology has advanced our understanding of intestinal physiology. Research integrating multi-omics technologies, including transcriptomics and proteomics, identified Hnf4g as a driver of major epigenetic, transcriptomic, and proteomic reprogramming during enterocyte differentiation, and a key regulator that sustains the balance between absorptive and secretory lineage-differentiated cells [216]. Additionally, using single-cell RNA sequencing (scRNA-seq) technology, research has identified a rare BEST4^+^ absorptive cell subtype in human colonic organoids and revealed that its differentiation is regulated by the Notch signaling pathway and the transcription factor SPIB. Subsequent pathway activity analysis indicated that this cell subtype may be involved in intestinal inflammation and diarrhea, which provides novel insights into intestinal physiology [217]. In addition, the integration of organoid technology and multi-omics analysis can provide insights into intestinal disease treatment. Through bulk transcriptomic and (scRNA-seq) analysis, research identified TNF-α as a key factor inducing ISC necrosis in CD patient-derived intestinal organoids. It also discovered that PGE2 signaling was found to repair TNF-α-induced ISC injury, implying that PGE2 may serve as a critical therapeutic target for restoring the intestinal barrier in CD [218].

In summary, the combination of intestinal organoid technology and new technologies such as microfluidics and gene editing provides a powerful research tool for in-depth analysis of intestinal physiological and pathological mechanisms and promotion of precision therapy. Although the application of these technologies in ISC research is still in its infancy, the advantages in reproducing the tissue microenvironment are evident. Thus, the combination of cutting-edge technologies and organoid culture technology is expected to reveal the mechanism of diseases more systematically and deeply, and to explore potential therapeutic targets.

### 5.3. Current Limitations of Intestinal Organoid Technology

Although intestinal organoid technology has greatly advanced research on intestinal physiology, pathology, and therapeutic development, it still has several limitations. First, standardized protocols for intestinal organoid culture are currently lacking. Variations in culture methods, passage numbers, and the composition of the culture medium often compromise experimental reproducibility. Second, intestinal organoids are predominantly composed of IECs arising from ISC differentiation. Even when co-culture is employed, it usually includes only one or a few cell types and also fails to recapitulate the complexity of in vivo intercellular signaling pathways and molecular interactions [219].

Microenvironmental factors, such as gut microbiota and their metabolites, oxygen gradients, and fluid shear stress, as well as their interactions, are all critical determinants of intestinal physiology and pathology, yet current organoid systems cannot fully replicate this intricate microenvironment. Beyond technical limitations, obtaining human intestinal tissue raises ethical challenges. The use of mucosal biopsy specimens further restricts the cellular diversity attainable in organoid cultures. The individual heterogeneity among patients demands large sample sizes to ensure the reliability of the result, which substantially increases both the difficulty of sample collection and the cost of intestinal organoid culture [220,221].

## 6. Conclusions

In conclusion, this review systematically reviews the research progress on ISCs and also summarizes the regulatory mechanism of niche cells, multiple signaling pathways, the ECM, and epigenetic factors on ISCs. The regulatory mechanisms of ISCs are highly complex, and these factors together constitute an intricate regulatory network. Different cellular states and distinct factors in the microenvironment are key variables that affect ISC activity. Currently, it remains unclear how to comprehensively evaluate the regulation of ISCs during the transition between different cellular states or microenvironments. Furthermore, it is still unknown which upstream niche-derived signals initiate ISC regulatory cascades that respond to ISC regulatory mechanisms and trigger the cascade of regulatory events.

At present, research on ISCs in disease is mainly focused on understanding the pathological mechanisms of CRC and IBD. Targeting ISCs has emerged as a potential therapeutic strategy, but its clinical application is still in the early stages. The rapid development of organoid technology has provided powerful tools for disease modeling and drug screening. This technology has significantly advanced precision therapy, especially in cancer research. In the future, a deeper understanding of the network regulation of ISCs within the microenvironment, combined with organoid co-culture systems and novel technologies such as microfluidic chips, is expected to systematically reveal the dynamic mechanisms of intestinal diseases and accelerate the identification of potential therapeutic targets.

## Figures and Tables

**Table 1:** The regulatory mechanism of signaling pathways on ISCs.

Signaling Pathway	Main Effectors	Key Mechanisms	Major Effect	Reference
Wnt/β-catenin pathway	β-catenin	When the pathway is activated, β-catenin enters the nucleus and binds to TCF/LEF, initiating gene transcription.	Driving ISC proliferation and self-renewal	[30,31,32,33,34,35,36,37,38]
Notch pathway	NICD	When the pathway is activated, NICD enters the nucleus and forms a complex with CSL, thus upregulating gene expression.	Regulating ISC differentiation	[39,40,41,42,43,44,45]
BMP pathway	Smad1/5/8	When the pathway is activated, phosphorylated Smad1/5/8 forms a transcriptional complex with Smad4, upregulating the differentiation-related gene expression.	Driving ISC differentiation	[46,47,48,49,50]
TGF-β pathway	Smad2/3	When the pathway is activated, Phosphorylated Smad2/3 translocates into the nucleus and forms a transcriptional complex with Smad4, upregulating the differentiation-related gene expression.	Limiting the excessive proliferation of the ISCs	[51,52,53,54,55,56,57]
Hippo pathway	YAP/TAZ	When the pathway is activated, YAP/TAZ is phosphorylated and retained in the cytoplasm for degradation, shutting down the ISC proliferation program.	Limiting the excessive proliferation of the ISCs	[58,59,60,61,62,63,64]
Hedgehog pathway	Gli	When the pathway is activated, Gli enters the nucleus and upregulates target genes, limiting the activity of the Wnt pathway.	Limiting the excessive proliferation of ISCs	[65,66,67,68,69,70]

## Data Availability

No new data were created or analyzed in this study.
